# Introducing Q Amyloid: a tool for automated PET‐based amyloid‐beta quantification

**DOI:** 10.1002/alz70862_110093

**Published:** 2025-12-23

**Authors:** Chiara Da Villa, Benedetta Marin, Francesco Piva, Lucia Maccioni, Manuela Moretto, Luigi Lorenzini, Mattia Veronese

**Affiliations:** ^1^ University of Padova, Padova Italy; ^2^ King’s College London, London UK; ^3^ Amsterdam University Medical Center (Amsterdam UMC), Amsterdam, North Holland Netherlands

## Abstract

**Background:**

The deposition of amyloid‐beta (Aβ) plaques is one of the earliest neuropathological hallmarks of Alzheimer's disease (AD) and precedes cognitive decline. Positron Emission Tomography (PET) is commonly adopted for *in vivo* Aβ detection and plays a pivotal role in preventing and enabling early intervention in AD. However, an accurate, efficient, and automated PET‐based pipeline to detect brain Aβ accumulation is still missing. The Q Amyloid platform will address this need. It will be tracer and scanning‐site agnostic, and enable the automated identification and quantification of Aβ burden through PET.

**Method:**

To develop and train the pipeline’s algorithms, a comprehensive dataset is being assembled. It combines several structural MRI and Aβ‐PET scans from public and private pre‐existing datasets, collected on individuals with mild cognitive impairment or dementia and healthy subjects. To ensure portability across Aβ radiotracers generally used in clinical and research settings, Aβ‐PET scans of ^11^C‐Pittsburgh compound B,^18^F‐florbetaben, ^18^F‐florbetapir, ^18^F‐flutemetamol are being included. The initial steps of the pipeline will involve data format harmonization, pre‐processing, and quality control. Aβ quantification will be performed using the Centiloid scale (Klunk et al., 2015), a quantitative 0‐100 global measure of Aβ load.

**Result:**

Taking the subject PET and MRI scans as input, Q Amyloid will be able to generate a subject‐specific report detailing the Centiloid numerical value and the status of Aβ pathology. This web‐based platform will be open‐access, dynamic, flexible and continuously updated with newly introduced methods for quantifying amyloidosis, including generative AI‐based approaches.

**Conclusion:**

Q Amyloid will be a valuable tool, both in supporting medical research to identify and track AD in its early stages and in providing pharmacodynamic biomarkers for developing anti‐amyloid therapies. Furthermore, it will facilitate the analysis of new clinical data, making it operative on an international basis. The platform will enable the maximization of methodology sharing and reusability in a decentralized manner. This project is supported by the cascading grant “Q Amyloid – Quantitative Amyloid Imaging” under PNRR ECS00000017 “THE ‐ Tuscany Health Ecosystem,” Spoke 6: “Precision Medicine & Personalized Healthcare” funded by the European Commission through the NextGeneration EU plan.

